# GAN-Augmented Naïve Bayes for identifying high-risk coronary artery disease patients using CT angiography data

**DOI:** 10.1038/s41598-024-73176-3

**Published:** 2024-10-07

**Authors:** Lei Zhang, Anandakumar Haldorai, Nithesh Naik

**Affiliations:** 1 School of Information and Electronic Engineering, Zhejiang University of Science and Technology, Hangzhou, 310023 Zhejiang China; 2 Key Laboratory of Biomedical Intelligent Computing Technology of Zhejiang Province, Hangzhou, 310023 Zhejiang China; 3 Department of Computer Science and Engineering, Sri Eshwar College of Engineering, Coimbatore, Tamilnadu India; 4 Department of Mechanical and Industrial Engineering, Manipal Institute of Technology, Manipal Academy of Higher Education, Manipal, Karnataka India

**Keywords:** Coronary artery disease, Cardiovascular, Coronary computed tomography angiography, Generative adversarial networks, GANs, Healthcare, Computational biology and bioinformatics, Cardiology, Health care, Mathematics and computing

## Abstract

Coronary artery disease (CAD) is one of the most common cardiovascular disorders affecting millions of individuals globally. It is the leading cause of mortality in both the wealthy and impoverished nations. CAD patients exhibit a wide range of symptoms, some of which are not evident until a major incident occurs. The development of techniques for early detection and precise diagnosis is heavily dependent on research. The proposed system introduces a novel approach, Generative Adversarial Networks Augmented Naïve Bayes (GAN-ANB), to classify high-risk CAD patients using Coronary Computed Tomography Angiography (CCTA) imaging data. The database included images from Coronary Computed Tomography Angiography (CCTA) records of 5,000 individuals. The developed GAN framework consists of a generator to generate synthetic patient profiles, and a discriminator to distinguish between genuine and synthetic profiles to improve the identification of high-risk CAD patients. Adding synthetic data to the training process allowed the discriminator to be utilized further to improve predictive modeling. The performance of the GAN-enhanced prediction model was assessed using accuracy, sensitivity, specificity, and area under the Receiver Operating Characteristic curve (ROC). The model exhibited an outstanding Dice Similarity Coefficient (0.91), Mean Intersection Over Union (0.90), recall (0.96), and precision (0.98) in differentiating between high-risk and low-risk individuals. The identification of high-risk patients with CAD is greatly enhanced by the integration of GANs with clinical and imaging data. ROC of 0.99 was achieved by the GAN-ANB model, which outperformed conventional machine learning models, was achieved using the GAN-ANB model. High cholesterol level, diabetes, and some CCTA-derived imaging characteristics, including plaque load and luminal stenosis, were among the major predictors. This method offers a powerful tool for early diagnosis and intervention, potentially leading to improved patient outcomes and lower healthcare expenditure.

## Introduction

Atherosclerosis narrows or blocks coronary arteries, limiting blood flow to the heart and resulting in Coronary Artery Disease (CAD), which is the leading cause of mortality globally. CAD ranges from asymptomatic atherosclerosis to acute coronary syndromes such as myocardial infarction and unstable angina. It typically affects men, smokers, the elderly, and individuals with a family history^1^. Symptoms include chest discomfort and shortness of breath, which can lead to heart attack and heart failure^2^. Despite therapeutic alternatives, CAD is a health concern worldwide, especially in low- and middle-income nations, emphasizing the need for prevention. In the United States alone, CAD kills approximately 370,000 people annually^3^.

Endothelial dysfunction causes CAD by enabling Low-Density Lipoprotein (LDL) to accumulate in the arterial wall, resulting in an inflammatory reaction. Monocytes enter the artery, differentiate into macrophages, and consume lipids, resulting in fatty streaks and foam cells^4^. These early lesions progressed to fibrous plaques over time. Plaque rupture can cause thrombosis and myocardial infarction^5^. As atherosclerosis advances, smooth muscle cells move from the arterial media to the intima and secrete extracellular matrix components that form a fibrous cap over the lipid core, resulting in a persistent plaque. On the other hand, chronic inflammation can destabilize plaques, leading to rupture and thrombosis, which can result in myocardial infarction or ischemia. Chronic coronary artery constriction can result in chronic angina, whereas rapid decreases in blood flow can cause Acute Coronary Syndrome (ACS)^6^.

A combination of modifiable and non-modifiable risk factors is frequently associated with CAD. It is possible to change the risk factors for obesity, diabetes, hypertension, smoking, eating poorly, and leading to an inactive lifestyle^7^. The development of CAD is influenced by a number of non-modifiable risk factors, including age, sex, ethnicity, and family history of the condition^8^. Significant risk factors for CAD have also been identified, such as low high-density lipoprotein levels, diabetes, hypertension, current smoking, and inadequate physical activity^9^. Important factors associated with CAD recurrence include genetic risk, age at first CAD incidence, high-density lipoprotein cholesterol concentration, clinical indications, and lifestyle decisions related to diet, physical activity, and smoking^10^. The identification and management of these risk factors are critical.

The global population is also expanding. It is estimated that there will be 8.5 billion people on Earth in 2030 and 9.7 billion in 2050^11^. Sub-Saharan Africa accounts for more than half of this growth. The global population of over 60 is growing, accounting for 1.4 billion of the total in 2030, 2.1 billion in 2050, and 12% of the global population in 2020^12^. As a result, although age-standardized mortality has declined globally due to improved prevention and treatment, the burden of non-communicable illnesses will continue to rise in terms of disability-adjusted life years and annual fatalities. This is especially true for the burden of cardiovascular disease, where it is anticipated that there will be 32,3 million fatalities from CAD in 2050 and over 22,2 million in 2030. Up to three major CAD events follow every CAD mortality, increasing the financial and societal costs associated with health care. The percentage of the world’s GDP allocated to healthcare will similarly rise, reaching 8.6% today to 8.9% in 2030 and 9.4 in 2050^13^.

A world with 50% fewer age-standardized cardiovascular deaths and a much lower age-standardized incidence of cardiovascular disease by 2050. However, a society in which everyone has an equal opportunity to achieve good cardiovascular health and, ultimately, premature cardiovascular death can be prevented globally. Ultimately, we envision a world in which everyone has a personal right to and responsibility for maintaining good cardiovascular health.

To determine the existence and severity of CAD, echocardiography, Coronary Computed Tomography Angiography (CCTA), and stress tests (pharmacological or treadmill) are frequently used^14^. As coronary angiography makes coronary artery blockages directly visible and measurable, it continues to be the gold standard for detecting coronary artery disease (CAD) (Patel et al., 2010).

With the potential to improve results and reduce side effects, genetic testing and biomarkers are increasingly being employed to customize medicines to the unique profiles of individual patients^15^.

The following are the novel findings of the proposed research, which uses Coronary Computed Tomography Angiography (CCTA) imaging data to identify high-risk patients for CAD using Generative Adversarial Networks (GANs) and Naïve Bayes.

i. The proposed study addresses the problem of sparsely annotated imaging data using GANs to produce realistic synthetic CCTA images. By adding value to the training dataset, the generalization and resilience of the classification model are strengthened.
ii. Adversarial training with GANs increases the CCTA image quality and resolution, which enhances the visibility of anatomical features for CAD diagnosis.
iii. GANs can help strengthen the resilience of the classification model by generating various samples to reduce overfitting and enhance generalization to fresh and unseen CCTA images.
iv. By combining the (GAN-ANB) classifier, the proposed model performs better in correctly identifying individuals at a high risk for CAD.

Section 2 outlines the literature review, followed by Sect. 3 outlining the proposed method, Sect. 4 presents the acquired results and discussion, and Sect. 5 concludes the paper.

## Literature review

Over the past several years, the identification and diagnosis of diseases have greatly benefited from the use of Machine Learning (ML) techniques. CNNs have the potential to inherit biases from the training set, which may result in skewed predictions that are not fair to all patient groups.

One major problem is maintaining balance in CNN-based predictions^16^.

Huang et al.^17^ demonstrated a contemporary method of healthcare prediction using machine learning, particularly the XGBoost model, to detect coronary artery disease risk factors. The association between putative risk factors and coronary artery disease was shown graphically using Shapely Additive Explanations (SHAP), which provided a fresh perspective on how to understand the results. The significance of various risk variables in the machine learning model was shown using the cover statistic to rank model covariates according to the overall prediction. To predict coronary vascular disease, Bajaj et al.^18^ employed a range of machine-learning approaches, including logistic regression, decision trees, random forests, and gradient-boosted trees. This study did not compare the performance of the machine learning models with conventional clinical risk assessment tools, which could have revealed important information about the added value of using ML in this situation, even though the gradient-boosted tree algorithm demonstrated promising results in predicting CAD outcomes.

The most accurate and reliable prediction of new-onset MACE is provided to patients with stable coronary artery disease using the age-biomarkers-clinical risk factor model, which integrates clinical risk factors with three biomarkers: lipocalin-2, A-FABP, and FGF-19. Due to variations in genetic, environmental, and lifestyle variables, models trained on data from a particular group or area cannot generalize well to other populations^19^.

Lipid-related indicators are used to predict CVD because of their known relationship with cardiovascular risk factors, including cholesterol levels. It may provide a snapshot of cardiovascular risk at a specific moment, but it does not take into account changes in lifestyle variables or medication adherence over time, which might affect long-term cardiovascular outcomes. This shortcoming highlights the necessity for dynamic risk assessment systems that incorporate longitudinal data and account for temporal changes in the risk variables. The prognostic value of lipid markers alone may be restricted^20^.

Furthermore, lipid indicators may not fully represent the complexities of cardiovascular risk, especially in various groups, or in those with metabolic syndrome or diabetes. These conditions can alter lipid metabolism and the risk profile for cardiovascular events, potentially reducing the reliability of lipid markers as standalone predictors.

Although Random Forest (RF) algorithms have shown promising results in CAD prediction, they have some significant drawbacks^21^. It is prone to overfitting, which is particularly problematic when working with high-dimensional datasets, or when the model is excessively complex with too many trees. This leads to the model capturing noise instead of underlying patterns, which reduces its generalizability to new unseen data.

Support Vector Machines (SVMs) have several obstacles. The performance of SVMs may be greatly impacted by the careful selection of the kernel function (linear, polynomial, radial basis function, etc.) and the tuning of hyperparameters, such as the kernel parameters and penalty parameters. Extensive grid search or cross-validation, which is computationally expensive and time consuming, is frequently used in this procedure^22^. SVMs perform poorly on large datasets owing to their computational complexity, which ranges from O(n^2^) to O(n^3^), where n denotes the number of data points. This makes them unsuitable for extremely large datasets^23^.

SVMs are sensitive to feature scaling and normalization, requiring all input features to be on a similar scale. This adds an extra preprocessing step that must be handled carefully to avoid introducing biases^24^. SVMs may have a bias in favor of the majority class, which makes it difficult to identify occurrences of the minority class, such as infrequent but important positive examples, in the prediction of CVD.

Recurrent Neural Networks (RNNs) predict CVD by analyzing both sequential data from patient records and time-series medical data^25^. However, RNNs experience vanishing and growing gradient challenges during training, particularly with extensive medical histories, limiting their ability to learn the long-term dependencies required for good CVD prediction. Training RNNs is computationally expensive and time-consuming because of their sequential structure, which processes each time step separately. This restriction makes it difficult to apply RNNs to large-scale healthcare datasets that are routinely utilized in CVD research. The requirement for vast amounts of labeled data for training RNNs is a considerable barrier to disease prediction.

Datasets used in CVD prediction frequently contain noise and class imbalances, with fewer patients with severe illnesses than healthy individuals. AdaBoost changes the weights to focus more on mispredicted instances, which might cause the model to overemphasize noisy or minority class data, potentially leading to overfitting and poor generalization to new cases^26^.

k-nearest neighbors (KNN) face unique obstacles when used to predict cardiovascular disease (CVD). A major concern is that it is susceptible to the curse of dimensionality, which is particularly challenging in CVD datasets with numerous variables, such as patient demographics, clinical assessments, test findings, and imaging data. In these high-dimensional domains, KNN’s distance measurements become less effective, making it harder to reliably identify related patients and resulting in poor performance^27^. KNN performance is largely dependent on and distance metric. A small value can make the model overly sensitive to noise and outliers inpatient data, while a large value can over smooth the model and miss important variations in patient conditions that are clinically relevant. The requirement for substantial parameter adjustment increases complexity and necessitates significant subject expertise and experimentation.

Bayesian networks are based on conditional probabilities and assumptions regarding variable independence, which may not always be valid in the complex multivariate nature of cardiovascular disorders^28^. Estimating parameters entails computing conditional probabilities from data, which may be resource-intensive and time consuming. Learning the network structure requires the investigation of a potentially large space for possible interactions among variables.

Specific technological restrictions were applied to each deep neural network (DNN) method employed for the prediction of CVD. To achieve high accuracy, a CNN requires a large amount of labeled training data, which is difficult to achieve^29^. Furthermore, CNN training requires considerable computational power and time. Training over lengthy sequences is difficult for RNN and lengthy Short-Term Memory Networks () because of vanishing and expanding gradient issues^30^. In particular, LSTMs are intricate and computationally taxing, consume large amounts of memory and computing power, and take longer to train than straightforward models. Moreover, it requires a large amount of sequential data that are properly labeled, which can be problematic in clinical situations.

Autoencoders encounter overfitting problems, particularly in cases in which the latent space is not appropriately regularized and may acquire complex and challenging characteristics^30^. The amount and quality of the input data they receive have a significant impact on their performance, and training them may be computationally costly.

Deep Belief Networks (DBNs) need meticulous hyperparameter tweaking, involve intricate and time-consuming training procedures, and perform poorly on very large datasets or deep architectures^31^. Similar to other deep learning models, they also have interpretability problems.

Training Generative Adversarial Networks (GANs) is difficult because of problems such as unstable training dynamics and mode collapse^32^. To produce realistic synthetic data, considerable processing power and a wide variety of training data are required. It is not always easy to assess the quality of the generated data; in many cases, manual examinations or specific metrics are required.

Multilayer Perceptrons (MLPs) struggle to handle high-dimensional data and intricate feature interactions found in medical datasets^33^. This requires careful feature engineering because in the absence of sufficient regularization, they are prone to overfitting. DNNs have the potential to predict CVD, but these technological issues need to be addressed to improve their accuracy and suitability for use in clinical settings^34^.

Ronneberger et al.^35^ proposed U-Net, which, while necessary for biomedical image segmentation, may suffer from more complicated datasets owing to the lack of sophisticated feature extraction capabilities. Alom et al.^36^ improved this with ResUNet, which incorporates residual connections to increase performance, although it can still struggle with extremely high-dimensional data or noisy images. Smith et al.^37^ addressed KNN, which is a simple technique but has limitations owing to its performance on complicated, high-dimensional datasets and its reliance on distance measurements, which can be less successful in capturing nuanced patterns.

Yang et al.^38^ proved the usefulness of CNNs for CVD classification. However, CNNs can be computationally expensive and require a large quantity of data to provide ideal results. Smith et al.^39^ proposed a GAN-Augmented Naïve Bayes (GAN ANB) model that achieves outstanding results but may require sophisticated implementation and adjustment to balance the generating and classification components.

### Dataset

Using Computed Tomography (CT) to provide high-resolution images of the coronary arteries, coronary CCTA is a noninvasive imaging technique. To make the coronary lumen and vessel walls more visible, an intravenous contrast dye injection was used. Less risk associated compared to traditional angiography, non-invasive. can be used to evaluate anomalies in the vessel wall and luminal anatomy in detail. useful in patients with low to intermediate risk to rule out CAD^40^. There were 206 patients in the collection, totaling 5.130 billion images. At 59.9 ± 9.4 years old, and 42.7% of the population was female. Patient information was gathered in a routine clinical setting. It has many characteristics that maintain the robustness of algorithms created in actual clinical applications. 64-slice CT scanners were used to gather cardiac CTA data, and images were reconstructed with a thickness of 0.50 mm. Table 1 lists the 11 cardiovascular structures identified in the datasets.

**Table 1:** Description of the CCTA dataset.

Dataset	CS	DA	IVC	LAA	LAW	PM	PML	PAA	PA	RVW	SVC
No. of patients	20	21	20	21	22	22	22	21	21	22	22
No.of 2D images	153	2005	288	365	806	869	454	355	309	1445	538

CS - Coronary Sinus. DA - Descending Aorta. IVC - Inferior Vena Cava. LAA - Left Atrial Appendage. LAW - Left Atrial Wall. PM - Papillary Muscle. PML - Posterior Mitral Leaflet. PAA - Proximal Ascending Aorta. PA - Pulmonary Aorta. RVW - Right Ventricular Wall. SVC - Superior Vena Cava.

Starting at the nadirs of all three aortic valve cusps, the PAA corresponded to the plane closest to the origin of the brachiocephalic artery. The DA began distal to the origin of the left subclavian artery and extended to the lowest axial disc. The vena cavae are venous veins that emerge from the right middle mediastinum, directly to the right of the trachea and PAA, and are empty into the right atrium. The PA included the left and right main pulmonary arteries. The CS exits the great cardiac vein at the left circumflex coronary artery, travels via the atrioventricular groove, and empties into the right atrium. Drawing the endocardial and ventricular walls allows one to calculate the RVW of the cardiac tissue.

**Table 2:** Baseline characteristics of the population.

Characteristic	Overall Population (*n* = 5000)
Demographics	
Age, mean (SD)	58.4 (12.3) years
Gender, n (%)	
Male	2,800 (56.0%)
Female	2,200 (44.0%)
Clinical history	
Hypertension, n (%)	2,500 (50.0%)
Diabetes Mellitus, n (%)	1,100 (22.0%)
Smoking Status, n (%)	
Current Smokers	1,200 (24.0%)
Former Smokers	1,000 (20.0%)
Non-Smokers	2,800 (56.0%)
Laboratory measures	
Cholesterol, mean (SD)	200.5 (45.7) mg/dL
LDL Cholesterol, mean (SD)	130.2 (35.4) mg/dL
HDL Cholesterol, mean (SD)	48.6 (12.3) mg/dL
Imaging data	
Calcium Score, mean (SD)	350.7 (420.9)
Coronary Artery Stenosis, n (%)	1,800 (36.0%)

As shown in Table 2, the baseline characteristics of the study population (*n* = 5,000) indicated a mean age of 58.4 years, with men accounting for 56% of the cohort. Clinically, half of the patients had a history of hypertension and 22% had been diagnosed with diabetes. Regarding smoking status, 24% were current smokers, 20% were past smokers, and 56% did not smoke. The laboratory results show an average total cholesterol level of 200.5 mg/dL, with LDL and HDL cholesterol levels averaging 130.2 mg/dL and 48.6 mg/dL, respectively. Imaging results showed that 36% of the patients had significant coronary artery stenosis, with a mean calcium score of 350.7.

Table 3 outlines the baseline characteristics of the male and female patients with CAD. Males showed higher rates of smoking history and myocardial infarction than females, which may contribute to higher overall risk levels. Conversely, females have a higher prevalence of post-menopausal status, a factor known to influence CAD risk. Hypertension is more common among females, while high LDL cholesterol levels are more prevalent in males. The p-values indicate the statistical significance of these differences, with smoking history and history of myocardial infarction showing significant sex disparities. Here, N represents the number of patients in each group and the p-value indicates statistical significance.

**Table 3:** Hypothetical values for baseline characteristics according to sex, including gender-specific assessments and risk factors.

Characteristic	Male (*N* = 2500)	Female (*N* = 2500)	Total (*N* = 5000)	*p*-value
Age (years)	Mean ± SD: 62 ± 8	Mean ± SD: 60 ± 7	Mean ± SD: 61 ± 7	0.02
Hypertension (%)	55%	60%	57.5%	0.03
Diabetes Mellitus (%)	30%	28%	29%	0.45
High LDL Cholesterol (%)	40%	35%	37.5%	0.12
Smoking History (%)	45%	20%	32.5%	< 0.001
History of Myocardial Infarction (%)	25%	15%	20%	< 0.001
Family History of CAD (%)	35%	30%	32.5%	0.15
Body Mass Index (BMI)	Mean ± SD: 27 ± 4	Mean ± SD: 26 ± 5	Mean ± SD: 26.5 ± 4.5	0.08
Post-Menopausal Status (%)	N/A	55%	55%	N/A
Atypical Symptoms (%)	20%	25%	22.5%	0.10

## Methodology

**Fig. 1:**
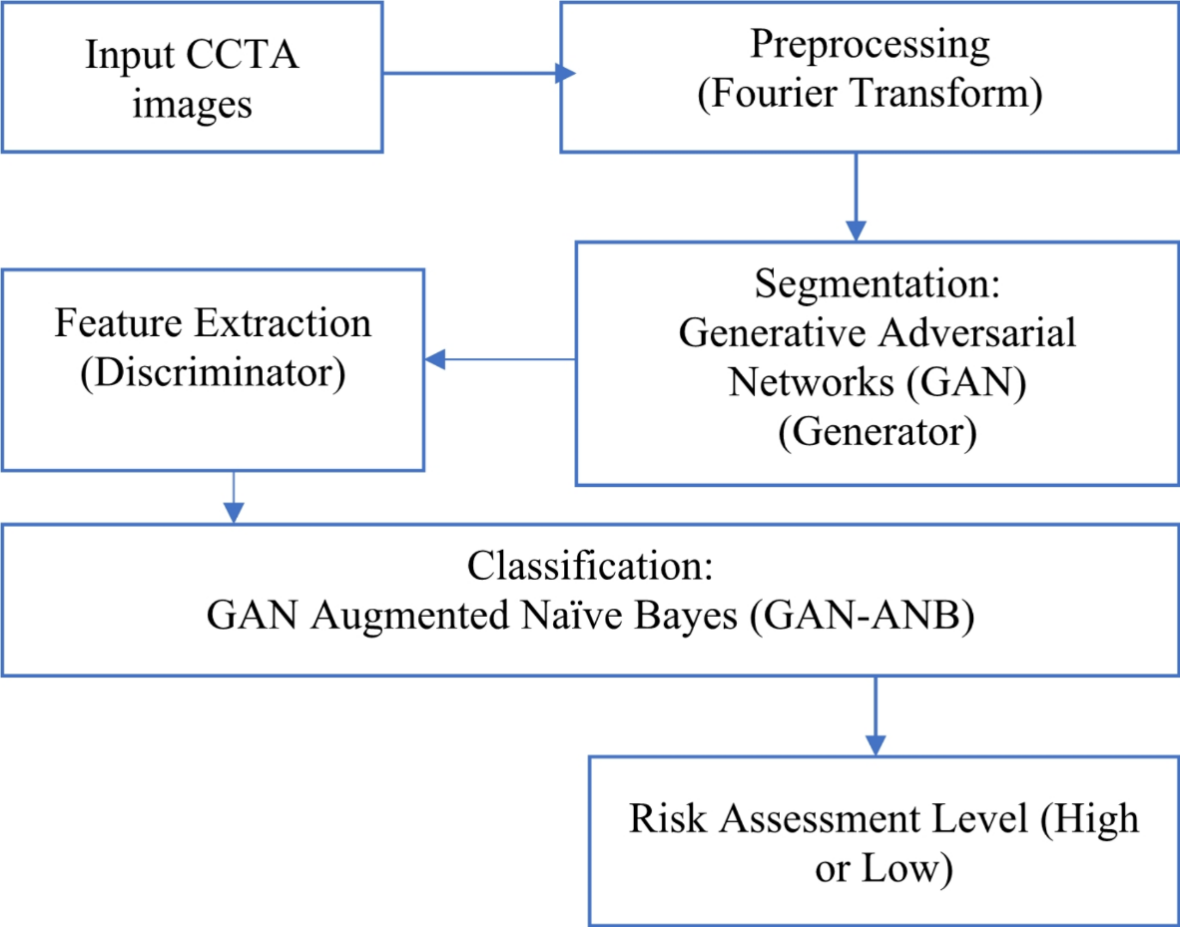
Block diagram of the proposed model to identify CAD.

The proposed system is illustrated in Fig. 1. Input of CCTA images is the first step in applying Generative Adversarial Networks (GANs) to CCTA imaging data to identify high-risk patients for CAD. Next, the images were preprocessed through image normalization and enhancement. The generator and discriminator, the two primary parts of the GAN, receive the previously processed images.

The discriminator distinguishes between actual and synthetic pictures to enhance the output of the generator, which produces synthetic CCTA images or enriches the original images to emphasize the CAD-relevant aspects. Once trained, the intermediate layers of the discriminator were used to extract rich and informative features from the real CCTA images. These features serve as input to the Naïve Bayes classifier, which models the probability distribution of the features for CAD and non-CAD cases to identify high-risk patients. The process of converting cardiovascular images from the spatial domain to the frequency domain for GAN-based segmentation preprocessing involves the use of Fourier Transform. Through this transformation, visual data may be altered in terms of their frequency components, which makes it possible to reduce noise or accentuate pertinent information. Certain features of cardiovascular architecture can be efficiently accentuated or suppressed using filters. Following filtering, the images were transformed back into the spatial domain using inverse Fourier Transform. The treated images exhibited decreased noise artifacts and improved structural features. These preprocessed images were used to train models for the precise segmentation of cardiovascular anatomy using Generative Adversarial Networks (GANs) as optimal inputs.

### Preprocessing

**Fig. 2:**
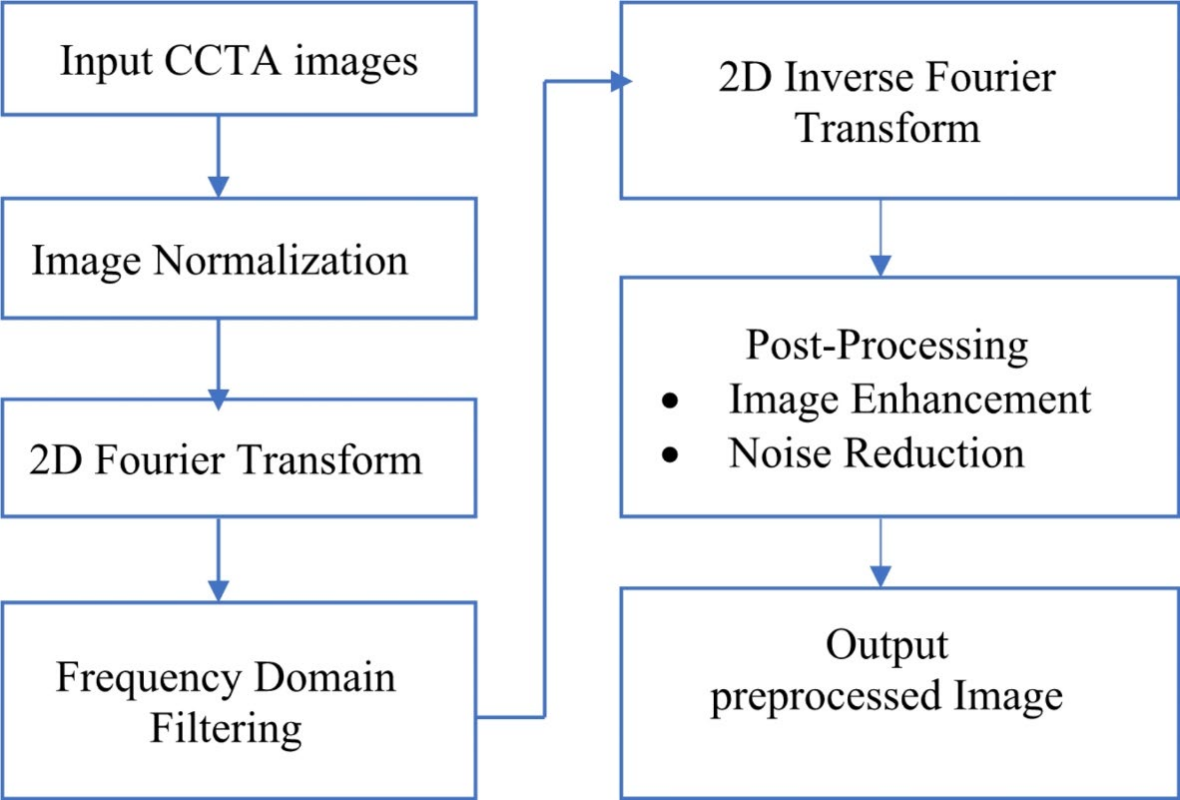
Preprocessing of CCTA images.

During acquisition, all types of noise, including electrical and photon noise, might impact CCTA images. The pre-processing steps are illustrated in Fig. 2.

As shown in Fig. 2, the input CCTA images were first captured and normalized to standardize intensity values. This is where the block diagram for preparing CCTA images using the Fourier Transform starts. Subsequently, a 2D Fourier Transform was applied to these images, shifting their spatial domains to their frequency domains. Different frequency-domain filtering techniques are used in the frequency domain to amplify or suppress particular frequency components. A 2D Inverse Fourier Transform was then used to return the filtered frequency-domain images to the spatial domain. To increase quality, the final images undergo post-processing procedures, including noise reduction and image enhancement.

The accuracy of diagnostic evaluations can be affected by noise artifacts, which can hide crucial anatomical information and be reduced with the use of preprocessing procedures. The Fourier Transform preprocessing of the cardiovascular images for GAN segmentation is described as follows: To transform an image from the spatial domain to the frequency domain, we applied a 2D Fourier Transform. By using the sine and cosine components to dissect an image, the Fourier Transform can be used to describe the CCTA image in terms of frequencies.

1 $$\:F(u,v)=\sum\:_{x=0}^{M-1}\sum\:_{y=0}^{N-1}f(x,y)\cdot \:{e}^{-j2\pi\:\left(\frac{ux}{M}+\frac{vy}{N}\right)}$$

where $\:f(x,y)$ is the pixel value at position $\:(x,y)$; *M* and *N* are the dimensions of the image, *u* and *v* are the frequency-domain coordinates, and *j* is the imaginary unit. The FFT shift moved the zero-frequency component to the center of the frequency-domain image for better visualization and processing.

2 $$\:{F}_{shifted}\left(FFTShift\right(F(u,v))$$

The filters in the frequency domain were applied to enhance the features and reduce noise.

3 $$\:{H}_{band-pass}(u,v)=\left\{\begin{array}{c}1\\\:0\end{array}\right.\genfrac{}{}{0pt}{}{if\:{D}_{1}\le\:\sqrt{{u}^{2}+{v}^{2}}}{otherwise}$$

The shifted Fourier Transform is multiplied using the chosen filter to enhance or suppress specific frequencies.

4 $$\:{F}_{filtered}(u,v)={F}_{shifted}(u,v)\cdot \:H(u,v)$$

The inverse 2D Fourier Transform is applied to convert the processed image back to the spatial domain.

5 $$\:{f}_{processed}(x,y)=IFFT2\left({F}_{inv-shifted}\right(u,v\left)\right)$$

Diagnostic accuracy and image quality can be compromised by CCTA image artifacts; such as motion artifacts resulting from patient movement or beam-hardening abnormalities caused by variations in tissue density. To ensure that the pixel values fall within a standard range, such as [0, 1] or [0, 255], the processed image is normalized.

6 $$\:{f}_{normalized}(x,y)=255\cdot \:\frac{{f}_{processed}(x,y)-min\left({f}_{processed}\right)}{max\left({f}_{processed}\right)-min\left({f}_{processed}\right)}$$

**Fig. 3:**
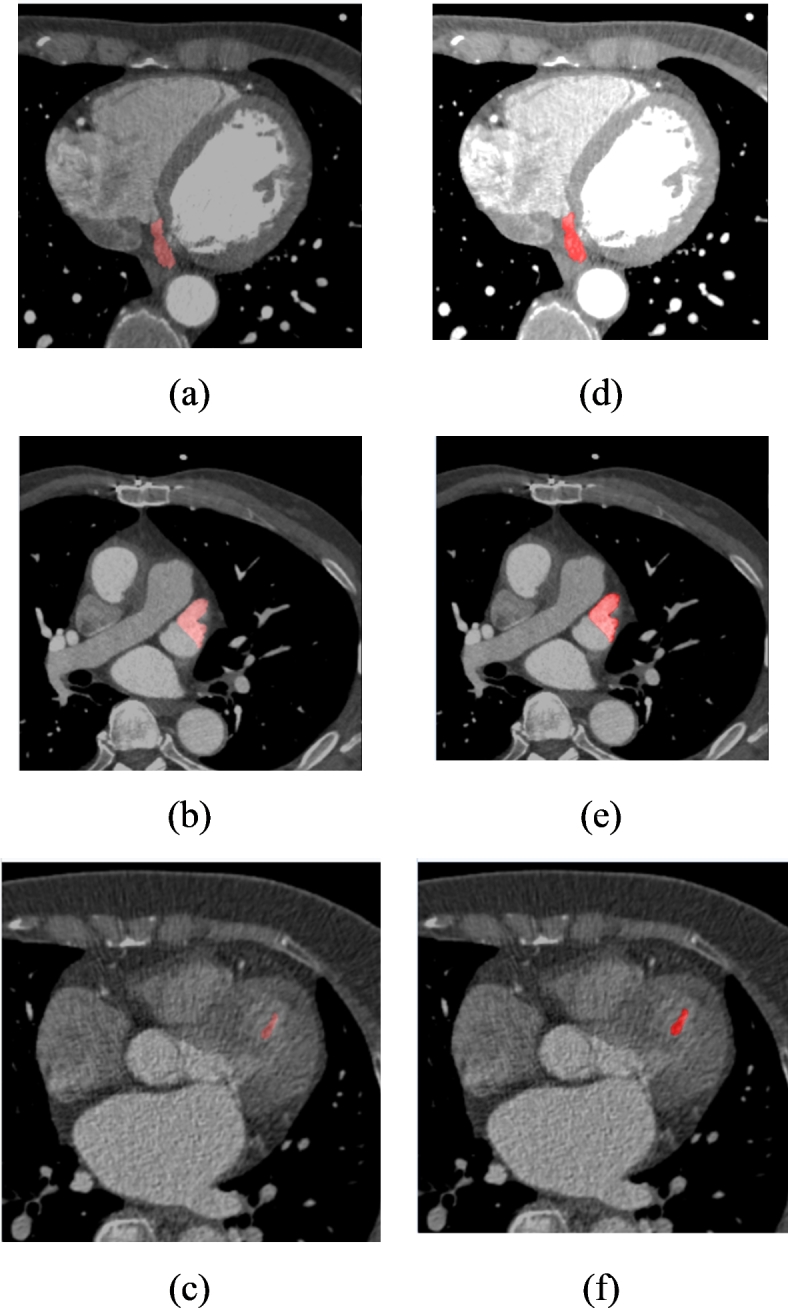
(**a**) to (**c**): Input CCTA images; (**b**)–(**f**) Preprocessed CCTA images by Fourier Transform.

**Fig. 4:**
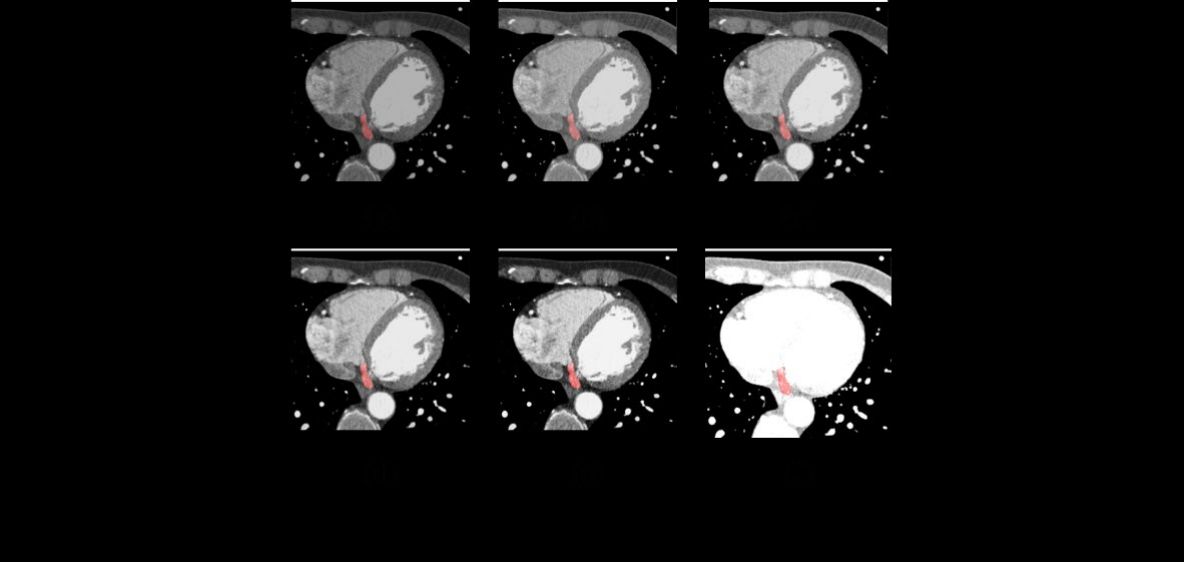
Preprocessed CCTA- CS images (CS) through the step-by-step process of Fourier Transform.

Figure 3 shows the high-risk CCTA images available in the dataset and the corresponding pre-processed images obtained through the Fourier Transform. The preprocessing steps of the CCTA images utilizing Fourier Transform are shown in Fig. 4. The input CCTA image is shown in Fig. 4(a), and includes noise, artifacts, and inconsistent image quality. In Fig. 4(b), the image is converted into the frequency domain using a 2D Fourier Transform, which symbolizes the frequency components of the image. Applying a low-pass filter decreases high-frequency noise, highlighting important structures, as shown in Fig. 4(c).

A high-pass filter that focuses on higher frequencies enhances the edges and fine details, as shown in Fig. 4(d). By allowing a certain range of frequencies to pass through, a band-pass filter was applied, as shown in Fig. 4(e), which balances the augmentation of both large structures and fine details. The preprocessed images are shown in Fig. 4(f), where they were transformed back into the spatial domain using 2D Inverse Fourier Transform. Preprocessing produces images with less noise and better visibility of important anatomical features, which are necessary for a precise diagnosis and analysis.

### Segmentation

After pre-processing, the enhanced cardiovascular images were fed into the GAN for segmentation. Using CCTA imaging data, Generative Adversarial Networks (GANs) are a revolutionary technique in medical image analysis for detecting individuals at high risk for CAD. A GAN is composed of two neural networks: a discriminator neural network and generator neural network. The participants received competitive training in parallel. The generator aims to produce realistic synthetic pictures of CCTA scans that resemble genuine images from high-risk patients, whereas the discriminator seeks to distinguish between real and synthetic images.

**Fig. 5:**
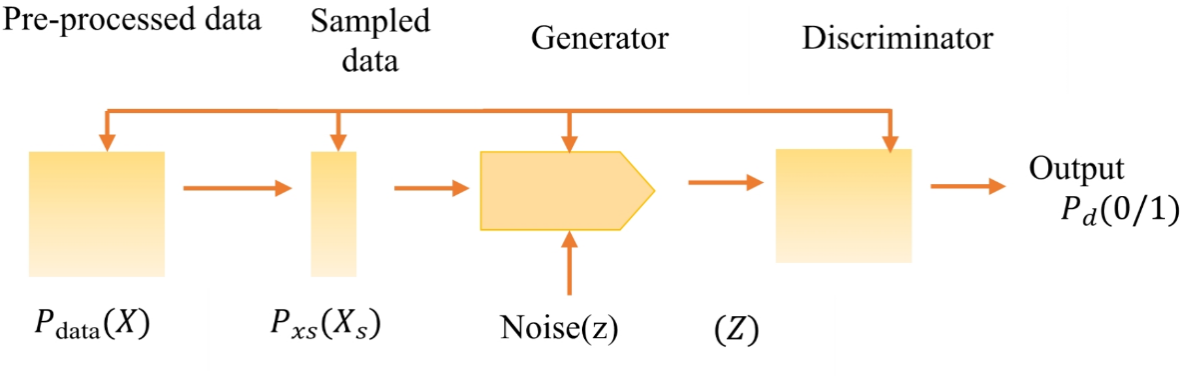
GAN for segmentation of CCTA imaging data.

As shown in Fig. 5, the generator network is accomplished through the following process: The input of generator *G* is random noise (z). Typically, a basic distribution, such as a Gaussian distribution, is used to generate this noise.

7 $$\:z \sim N\left(\text{0,1}\right)$$

Generator *G* consists of multiple layers of neural network units. These layers are used to transform the input noise *z* into a higher-dimensional representation, which can be interpreted as an image.

The generator *G’s*$\:{\theta\:}_{G}$ parameters of the generator G are tuned during the training phase to obtain a mapping between the output image space and the input noise space. The gradients from discriminator *D* are utilized to update $\:{\theta\:}_{G}$ throughout this backpropagation learning phase within the GAN framework.

The final output of the generator network * is* a synthetic CCTA image $\:\widehat{X}$. This image is generated in such a way that it should resemble real CCTA scans of high-risk patients in terms of key features and patterns.

Discriminator *D’s* task is to reduce the degree to which the produced images $\:\widehat{X}\:$ can be distinguished from the actual CCTA images *X* by the generator. To deceive D into thinking that the pictures are genuine, G wants to create $\:\widehat{X}\:$ that is sufficiently realistic.

The generator *G* is updated during training using the gradients obtained from the feedback of discriminator *D*. Iteratively, this adversarial process enhances *G’s* capacity of G to produce increasingly accurate and lifelike synthetic CCTA images. The following elements are part of the GAN framework.

### Generator(G)

The generator network *G* learns to generate synthetic CCTA images $\:\widehat{X}$ from random noise *z.*

8 $$\:\widehat{X}=G(z;{\theta\:}_{G})$$

where $\:{\theta\:}_{G}$ represents the parameters of the generator network.

The transformation includes many layers, such as activation functions, batch normalization layers, transposed convolutional layers, and fully connected layers. The following is a representation of the overall framework:

9 $$\:\widehat{X}={f}_{L}\left({f}_{L-1}\right(\cdot\cdot\cdot{f}_{2}\left({f}_{1}\right(z))))$$

where $\:{f}_{L}$ denotes the function represented by the l^th^ layer in the network and *L* is the number of layers.

### Discriminator (D)

Discriminator network *D* aims to distinguish between real CCTA images *X* from high-risk patients and synthetic images $\:\widehat{X}$.

10 $$\:D(X;\:{\theta\:}_{D})={g}_{K}\left({g}_{K-1}\right(\cdot\cdot\cdot{g}_{2}\left({g}_{1}\right(X\left)\right)\left)\right)$$

where $\:{\theta\:}_{D}$ represents the parameters of the discriminator network, and $\:{g}_{K}$ denotes the function represented by the k^th^ layer in the network, and *K* denotes the number of layers.

11 $$\:D(X;\:{\theta\:}_{D})\approx\:\:1\:\to\:\:Probability\:that\:X\:is\:real$$

12 $$\:D(\widehat{X};\:{\theta\:}_{D})\:\approx\:\:0\to\:\:Probability\:that\:\widehat{X}\:is\:real$$

#### Adversarial training

The training process involves minimizing the following objective function, which balances the generator’s goal of fooling the discriminator and the discriminator’s goal of correctly distinguishing real images from synthetic images.

13 $$\:\underset{G}{\text{min}}\underset{D}{\text{max}}{\mathbb{E}}_{X\sim{P}_{data\left(X\right)}\left[log\:D\left(X\right)\right]}+{\mathbb{E}}_{z\sim{p}_{z\left(Z\right)}\left[log(1-D(G\left(z\right)\left)\right)\right]}$$

where $\:{P}_{data\left(X\right)}$ is the distribution of real CCTA images from high-risk patients and $\:{p}_{z\left(Z\right)}$ is the prior distribution of the input noise, z.

Once trained, generator *G* can produce synthetic CCTA images $\:\widehat{X\:}$that resemble CCTA images of high-risk patients. Clinicians can detect the characteristics or trends that point to CAD risk factors, including stenosis, plaque accumulation, or other anomalies, by examining the images.

### Loss functions for training

Different loss functions are used for the training of the discriminator and the generator.

Generator Loss is as follows.

14 $$\:{L}_{G}\:=\:-\:{\mathbb{E}}_{z\sim{p}_{z\left(Z\right)}\left[log\left(D\right(G\left(z\right))\right]}$$

Discriminator Loss is as follows.

15 $$\:{L}_{D}=-{\mathbb{E}}_{X\sim{P}_{data\left(X\right)}\left[log\:D\left(X\right)\right]}+{\mathbb{E}}_{z\sim{p}_{z\left(Z\right)}\left[log(1-D(G\left(z\right)\left)\right)\right]}$$

### Gradient descent updates

Training involves updating the parameters *G* and *D* using gradient descent. The update rule for the discriminator is as follows.

16 $$\:{\theta\:}_{D}\leftarrow\:{\theta\:}_{D}+\eta\:{\nabla\:}_{{\theta\:}_{D}}{L}_{D}$$

where $\:\eta\:$ is the learning rate.

For the generator, the update rule is as follows.

17 $$\:{\theta\:}_{G}\leftarrow\:{\theta\:}_{G}+\eta\:{\nabla\:}_{{\theta\:}_{G}}{L}_{G}$$

CAD risk assessment based on CCTA scans may be more accurate using GANs for this work because of their capacity to recognize intricate patterns and variability in medical imaging data. This approach aims to help physicians with early identification and individualized treatment planning for high-risk CAD patients. This is an inventive use of deep learning in the healthcare industry.

### Training algorithm

The GAN training algorithm involves iterating over the following steps:

- Step 1: Sample a mini-batch of real CCTA images $\:{\left\{{X}^{\left(i\right)}\right\}}_{i=1}^{m}$ from the real data distribution $\:{p}_{data}\left(X\right).$
  Step 2: Sample a mini-batch of noise vectors $\:{\left\{{z}^{\left(i\right)}\right\}}_{i=1}^{m}$ ​ from prior distribution $\:{p}_{z}\left(z\right).$
  Step 3: Generate synthetic images $\:{\left\{{\widehat{X}}^{\left(i\right)}\right\}}_{i=1}^{m}$ using the generator G, as shown in Eq. (1).
  Step 3: Compute the discriminator loss $\:{L}_{D}$​ using the real images and generated images, as shown in Eq. (5).
  Step 4: Update the discriminator parameter $\:{\theta\:}_{D}$ using gradient descent, as shown in Eq. (6).
  Step 5: Compute the generator loss $\:{L}_{G}$​ using the generated images, as shown in Eq. (7).
  Step 6: Update the generator parameters $\:{\theta\:}_{G}$ using gradient descent, as shown in Eq. (8).

In a GAN, generator *G* gains the ability to produce artificial CCTA images by converting random noise *z* into images *X* using a neural network trained with the adversarial feedback of discriminator D. To generate realistic pictures, optimization entails reducing the generator’s loss and optimizing the discriminator’s capacity to discern between genuine and artificial images. Using this adversarial training procedure, the generator can understand the intricate patterns and characteristics found in actual CCTA images from high-risk CAD patients.

### Feature extraction

The discriminator *D* consists of multiple layers, and $\:{\upvarphi\:}\left(x\right)\:$denotes the output of the intermediate layers. Let $\:{X}_{real}\:$be the actual CCTA-segmented image from the generator. These images pass through the discriminator to obtain intermediate features. $\:D\left(x\right)\:$is composed of *L* layers. Then,

18 $$\:D\left(x\right)={D}_{L}^\circ\:\:{D}_{L-1}^\circ\:\cdot\cdot\cdot^\circ\:\:{D}_{1}\left(x\right)$$

Where $\:{D}_{i}$​ denotes the i^th^ layer of the discriminator. The feature representation $\:{\upvarphi\:}\left(x\right)\:can\:be$ obtained from any layer *i*:

19 $$\:{\upvarphi\:}\left(x\right)={D}_{i}^\circ\:\:{D}_{i-1}^\circ\:\cdot\cdot\cdot^\circ\:\:{D}_{1}\left(x\right)$$

To obtain the feature representations the following equation is used.

20 $$\:{\text{F}}_{real}={\upvarphi\:}\left({x}_{real}\right)$$

where $\:{\text{F}}_{real}$ is the matrix of feature vectors for the real images.

### Pseudocode for Discriminator-based feature extraction

The procedure for extracting features from CCTA images using a GAN discriminator is described in the following pseudocode:

# Define GAN structure.

initialize generator G.

initialize discriminator D.

# Train GAN.

for each epoch in training_epochs:

for each batch in the dataset:

# Train discriminator D with real and fake images.

real_images = get_real_images(batch).

fake_images = G(generate_random_noise(batch_size)).

train D with real_images and fake_images.

# Train generator G to fool discriminator D.

fake_images = G(generate_random_noise(batch_size)).

train G to fool D with fake_images.

# Use the trained discriminator D to extract features.

for each preprocessed_image in the dataset:

features = D.extract_features(preprocessed_image).

store features in feature_list.

### Classification

High-quality features from the GAN discriminator help the GAN Augmented Naïve Bayes (GAN-ANB) Bayes to perform better, as these features are likely to be less correlated. The extracted features, $\:{\text{F}}_{real}$ is used by the Gaussian Naive Bayes classifier to classify these features. Naïve Bayes provides a simple yet efficient method that helps with illness detection and clinical decision-making by assuming the conditional independence of features given the class label. To determine a class’s posterior probability $\:{C}_{k}$, given the features $\:{x}_{1},{x}_{2},\cdot\cdot\cdot,\:{x}_{n}\:$are as follows:

21 $$\:P(\left.{C}_{k}\right|{x}_{1},{x}_{2},\cdot\cdot\cdot,\:{x}_{n})=\frac{P\left({C}_{k}\right){\prod\:}_{i=1}^{n}P({{x}_{i}\left|C\right.}_{k})}{P({x}_{1},{x}_{2},\cdot\cdot\cdot,\:{x}_{n})}$$

where:

- $\:P\left(\left.{C}_{k}\right|{x}_{1},{x}_{2},\dots\:,{x}_{n}\right)$is the posterior probability of class ​ $\:{C}_{k}\:$given the features $\:{x}_{1},{x}_{2},\:\dots\:,\:{x}_{n}.$
- $\:P\left({C}_{k}\right)$is the prior probability of class $\:{C}_{k}$​.
- $\:P\left({{x}_{i}\left|C\right.}_{k}\right)\:$is the conditional probability of feature $\:{x}_{i}$ given class $\:{C}_{k}.$
- $\:{P(x}_{1},{x}_{2},\dots\:,{x}_{n})$ is the evidence probability, which acts as a normalization factor.

GAN-ANB is suitable for discrete features and is typically used with counts or frequencies.

22 $$\:P\left(\left.{x}_{i}\right|{C}_{k}\right)=\frac{count({x}_{i},\:{C}_{k})+\alpha\:}{\sum\:_{{x}^{{\prime\:}}}count({x}^{{\prime\:}},\:{C}_{k})+\alpha\:}$$

where $\:count({x}_{i},\:{C}_{k})\:$is the count of features $\:{x}_{i}$​ in class $\:{C}_{k}$​ and $\:\alpha\:\:$is a smoothing parameter used to handle unseen features.

Calculate the posterior probability of each class $\:{C}_{k\:}$based on the training set.

23 $$\:P\left({C}_{k}\right)=\frac{No.of\:instance\:of\:class\:{C}_{k}}{Total\:no.of\:instances}$$

$\:\:\:\:\:\:P\left(\left.{x}_{i}\right|{C}_{k}\right)\:es$timate the conditional probabilities for each feature $\:{x}_{i}$, ​ given each class $\:{C}_{k}$​ based on the training data. For a new CCTA image with features $\:{x}_{1},{x}_{2},\dots\:,{x}_{n}$ calculate the posterior probability is calculated as follows:

24 $$\:P(\left.{C}_{k}\right|{x}_{1},{x}_{2},\cdot\cdot\cdot,\:{x}_{n})\propto\:\:P\left({C}_{k}\right){\prod\:}_{i=1}^{n}P({{x}_{i}\left|C\right.}_{k})$$

Normalize using the evidence probability for all classes to obtain $\:P(\left.{C}_{k}\right|{x}_{1},{x}_{2},\cdot\cdot\cdot,\:{x}_{n}).$ Assign the class label $\:\widehat{y}\:$to $\:arg\underset{k}{\text{max}}P(\left.{C}_{k}\right|{x}_{1},{x}_{2},\cdot\cdot\cdot,\:{x}_{n})\:$, the class with the largest posterior probability. Assessing the degree of blockage, narrowing, or plaques in the coronary arteries is a part of the treatment of CAD. Signs such as enlarged ventricles, poor ejection fraction, or aberrant heart wall movement are considered when classifying heart failure.

### Pseudocode of GAN-ANB classification

The pseudocode outlines the process of classifying CCTA images using GAN-ANB, incorporating feature extraction from the GAN discriminator.

Convert features and labels into arrays.

features_array = convert feature_list to an array.

labels_array = convert labels to array.

# Divide data into sets for testing and training.

train_features, test_features, train_labels, test_labels = split dataset into train and test sets.

# Training the GAN-ANB classifier.

naive_bayes = initialize GAN-ANB classifier.

naive_bayes.fit(train_features, train_labels).

# Evaluate classifier.

predicted_labels = GAN_ANB.predict(test_features)

accuracy = compute accuracy (test_labels, predicted_labels).

To generate personalized risk predictions for CAD using the GAN-ANB model, a multistep approach was employed. First, relevant patient features, including demographic information and CAD indicators, were extracted from the clinical and imaging data. The GAN component is then used to augment the dataset by generating synthetic data that simulate various CAD manifestations, which helps balance and enhance the dataset. This augmented data, combined with real patient data, is fed into the Naïve Bayes classifier. The classifier calculates the probabilities of high- and low-risk for CAD based on the patient’s features. Risk score *S* was computed using the following formula:

25 $$\:S=\frac{P(Y=1|X)}{P(Y=0|X)}$$

where $\:(Y=1|X)$ denotes the probability of being high risk given features *X*, and $\:P(Y=0|X)$ is the probability of being low risk. Alternatively, if the model provides a direct risk score, it is represented as $\:S=f(X;\theta\:)$, where *f* is the function of the GAN-ANB model, and *θ* represents the trained parameters. This process allows the GAN-ANB model to generate nuanced, sex-specific risk scores and recommendations, thereby improving personalized risk assessment and management for CAD by incorporating both real and synthetic data to capture a comprehensive risk profile for each patient.

The proposed study employed a thorough strategy to identify high-risk categories. Patients with substantial stenosis, defined as a coronary artery narrowing of ≥ 70%, indicating serious blockage, were included in this group. Also taken into account of patients whose CAD affects two or more coronary arteries, indicating a broad illness. Additionally, high-risk individuals are identified by the important features of ACS-prone plaques, such as positive remodeling, wherein the plaques induce the artery to grow outward, and spotty calcification, which is an uneven, patchy calcification that increases plaque instability. High-risk individuals also have noticeable clinical symptoms or a history of significant CAD events such as myocardial infarction or recurrent angina. This comprehensive description enabled the proposed method to concentrate on patients with the most severe and unstable CAD characteristics.

### Results and discussions

The proposed model leverages a combination of Generative Adversarial Networks (GAN) and GAN-ANB. To guarantee quality and consistency, the dataset underwent extensive pre-processing. The training and testing sets of the dataset were separated to enable a thorough assessment of model performance. The output of the GAN is used to augment the training data, which enhances the variability and richness of the dataset. This augmented dataset is then fed into a Naïve Bayes classifier, which is selected for probabilistic classification jobs owing to its efficiency and simplicity.

**Fig. 6:**
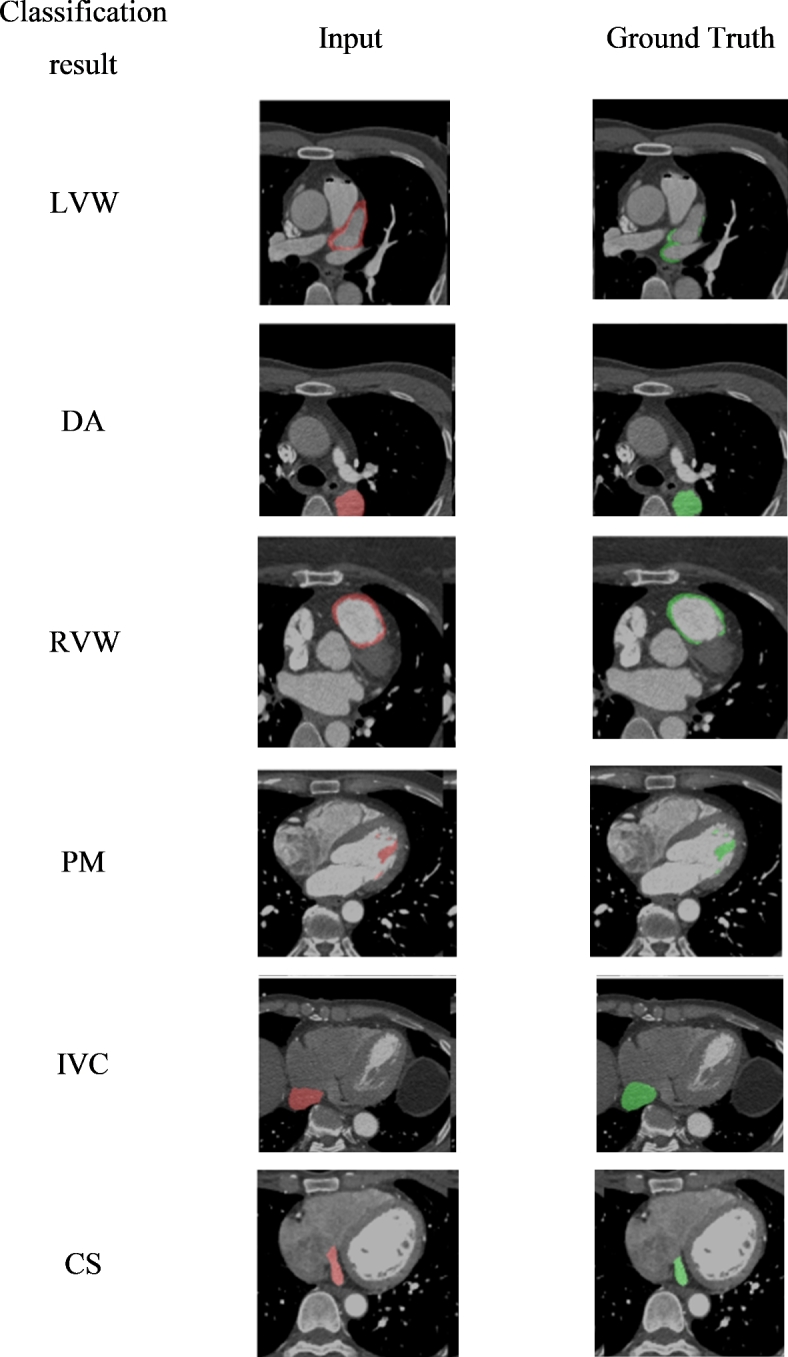
CVD classification results of the proposed model.

 The combined approach of GAN for data augmentation and Naïve Bayes for classification demonstrates promising results, showing improved classification performance owing to the enhanced dataset diversity provided by GAN, as shown in Fig. 6. This hybrid model offers a novel approach to medical image classification, potentially aiding in more accurate and early diagnosis of cardiovascular diseases. To ensure that the model is accurate and dependable in its predictions, the following performance metrics offer a thorough assessment of the model’s performance in categorizing and segmenting the CVD images.

### Dice similarity coefficient (DSC)

The DSC is a statistical metric that compares the similarity between two sets of data. It is very useful in image segmentation to determine the overlap between the expected segmentation and ground truth.

26 $$\:DSC=\frac{2\times\:\left|X\cap\:Y\right|}{\left|X\right|+\left|Y\right|}$$

A DSC of one indicates complete overlap, whereas a DSC of zero indicates no overlap.

### Mean intersection over union (mIoU)

mIoU is another method for assessing image segmentation performance. It computes the average IoU for all classes in multiclass segmentation.

27 $$\:mIoU=\frac{\left|X\cap\:Y\right|}{\left|X\cup\:Y\right|}$$

Higher mIoU values indicate an improved segmentation performance. An IoU of one indicates complete segmentation.

### Recall

Recall, also known as the sensitivity or true positive rate, is the proportion of genuine positives that the model accurately detects.

28 $$\:Recall=\frac{True\:Positives}{True\:Positives+False\:Negatives}$$

Recall ranged from 0 to 1, with 1 indicating that all affirmative cases were properly recognized.

### Precision

Precision, which is often referred to as the Positive Predictive Value, is the proportion of correctly predicted positives.

29 $$\:Precision=\frac{True\:Positives}{True\:Positives+\:False\:Positives}\:$$

**Fig. 7:**
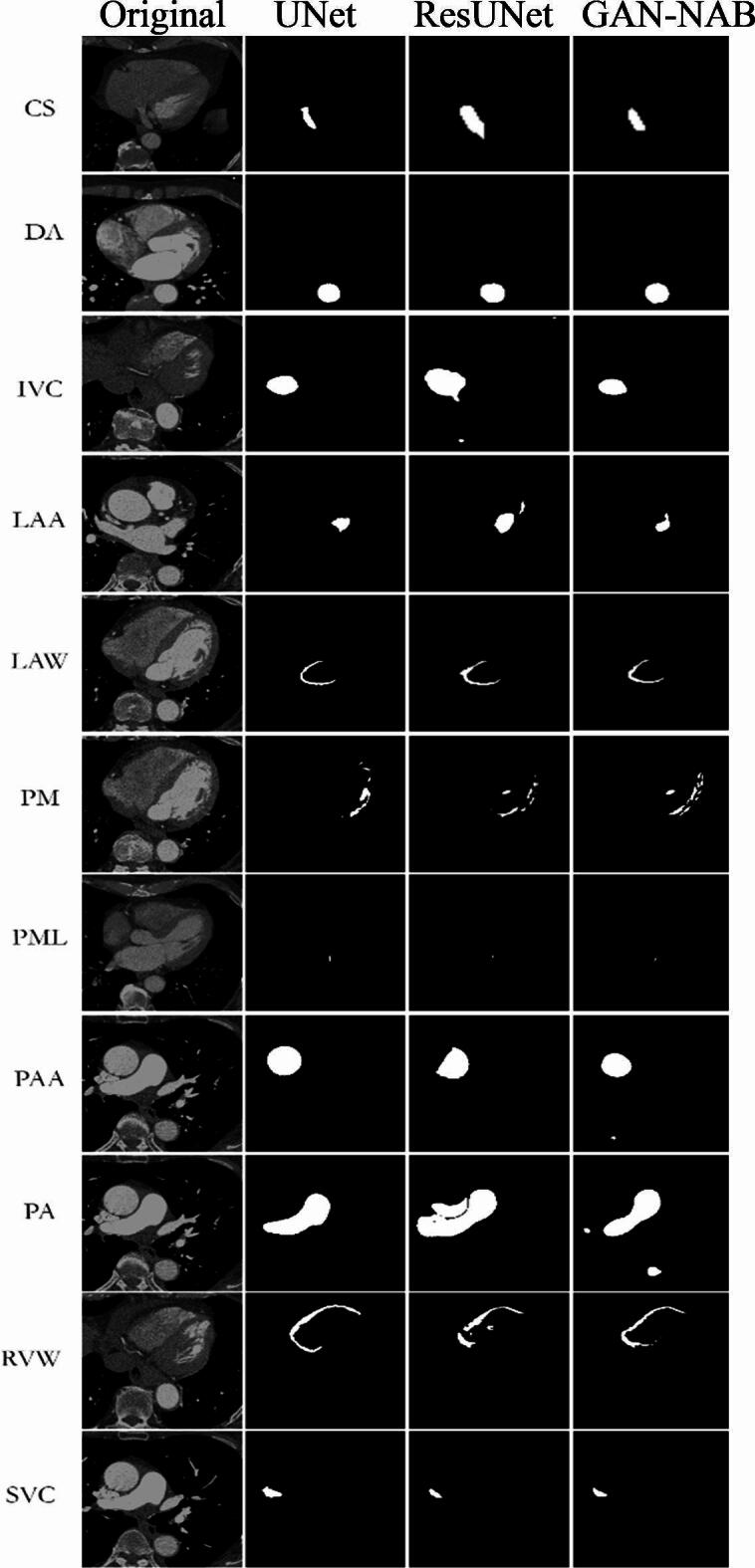
Comparison of obtained segmentation results with competitive methods.

As shown in Fig. 7, the segmentation results obtained for different cardiovascular structures using the proposed technique were highly promising. The performance metrics, including the Dice Similarity Coefficient (DSC), recall, precision, and Mean Intersection over Union (mIoU), show how well the model distinguishes and divides important cardiovascular structures. Table 4 shows the technique’s good segmentation performance for the left ventricle, with a DSC of 0.95, mIoU of 0.92, recall of 0.94, precision of 0.96, and accuracy of 0.93. With a DSC of 0.90, mIoU of 0.88, recall of 0.89, precision of 0.91, and accuracy of 0.89, the right ventricle also displayed impressive findings. With a DSC of 0.93, mIoU of 0.90, recall of 0.92, precision of 0.94, and accuracy of 0.91, the myocardium likewise demonstrated strong performance. Good performance metrics were also demonstrated by the left and right atria, demonstrating the dependability of the model in segmenting these structures. A DSC of 0.92, a mIoU of 0.89, a recall of 0.91, a precision of 0.93, and an accuracy of 0.90 were attained by the left atrium, and a DSC of 0.89, a mIoU of 0.86, a recall of 0.88, a precision of 0.90, and an accuracy of 0.87 was attained by the right atrium. These findings imply that the suggested method is quite successful at differentiating between different cardiovascular structures, which makes it a useful tool for predicting CVD and may contribute to earlier and more precise diagnosis.

**Table 4:** Segmentation findings of several cardiovascular structures obtained using the proposed methodology.

Dataset	GAN-ANB			
	DSC	mIoU	Recall	Precision
CS	0.88	0.87	0.97	0.99
DA	0.94	0.96	0.94	0.98
IVC	0.96	0.90	0.97	0.99
LAA	0.89	0.88	0.98	0.99
LAW	0.82	0.84	0.95	0.99
PM	0.84	0.87	0.92	0.97
PML	0.81	0.79	0.94	0.97
PAA	0.96	0.98	0.98	0.99
PA	0.99	0.98	0.99	0.98
RVW	0.98	0.92	0.99	0.98
SVC	0.95	0.94	0.98	0.99
Average	0.91	0.90	0.96	0.98

**Fig. 8:**
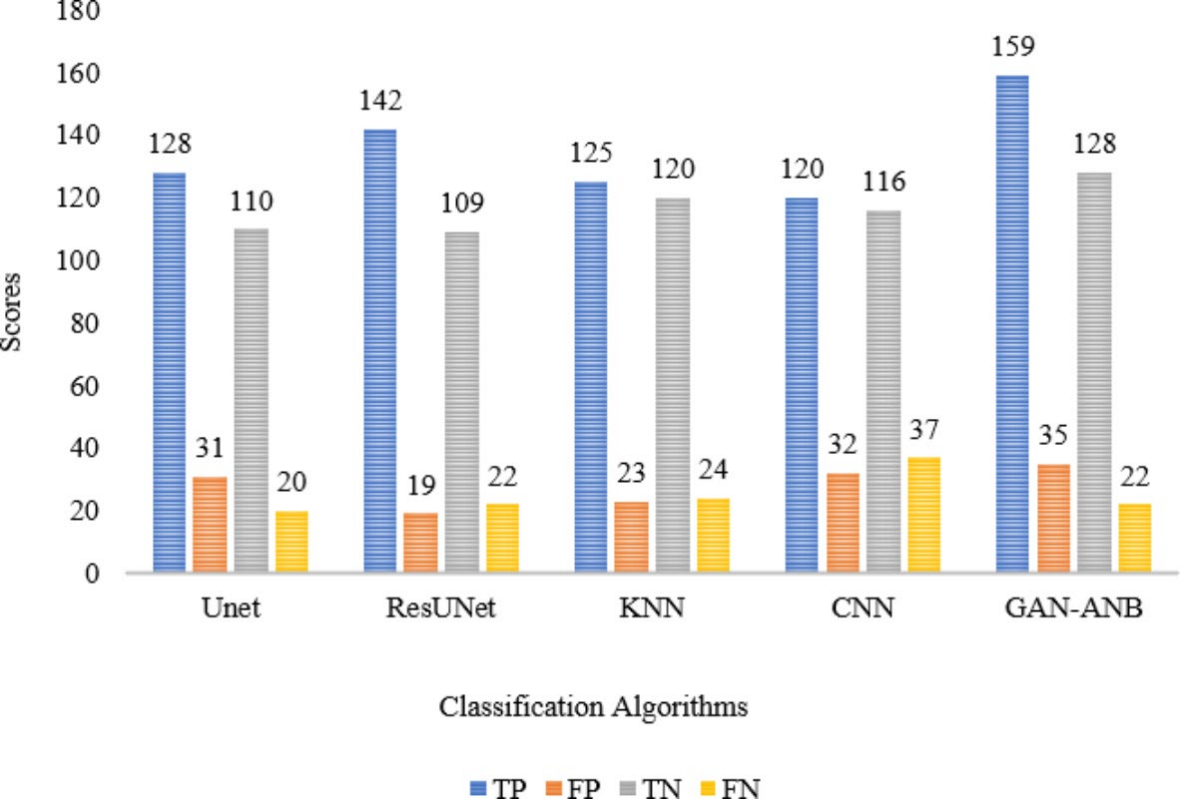
Comparison of CVD classification results with competitive methods.

As shown in Fig. 8, GAN-ANB exhibits the best overall performance across most metrics, making it a strong choice for CVD classification. As shown in Table 5, a comparison of several methods for classifying CVD reveals their unique strengths and limitations. UNet provides a solid framework for segmentation tasks, although it cannot capture all complicated patterns in the data^35^. ResUNet improves on UNet by including residual learning, which results in higher accuracy; however, it still struggles with very complex images^36^. K-Nearest Neighbors (KNN) have inferior performance, most likely because of their simplicity and limited capacity to handle high-dimensional data adequately^37^. Convolutional Neural Networks (CNN) provide robust results through deep learning, but they are computationally expensive and require a large amount of training data^38^. The GAN-ANB model combines the generating and classification approaches, leading to considerable accuracy improvements^39^.

**Table 5:** Comparison of CVD classification methods.

Model	DSC	mIoU	Recall	Precision
UNet^36^	0.8	0.78	0.75	0.85
ResUNet^37^	0.85	0.83	0.8	0.87
KNN^38^	0.6	0.55	0.6	0.62
CNN^39^	0.88	0.86	0.85	0.89
GAN-ANB^40^	0.89	0.88	0.86	0.93
Proposed Method	0.91	0.90	0.96	0.98

The proposed method outperformed all the other methods, delivering greater performance across measures. However, it may increase the implementation complexity and require careful calibration of both GAN and Naïve Bayes components.

**Fig. 9:**
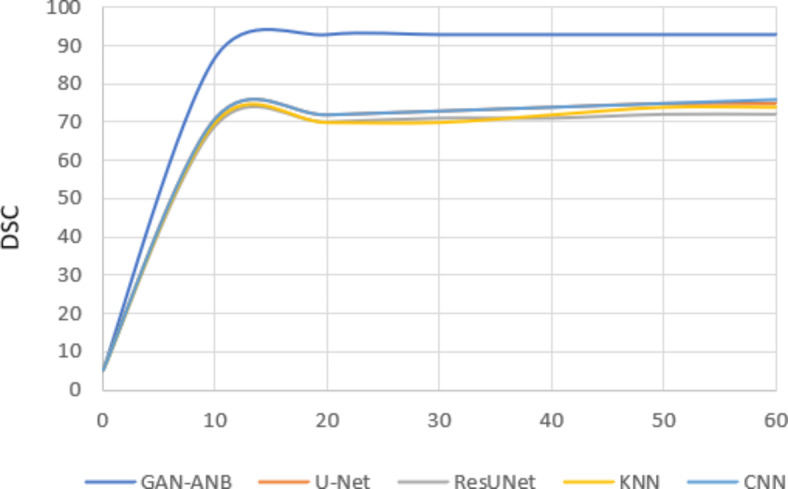
Comparison of DSC.

GAN-ANB had the highest DSC value of 0.91, indicating a good balance in segmenting CCTA images. CNN (0.76), U-Net (0.75), and KNN (0.74) showed strong but slightly lower performance. ResUNet scored the lowest at 0.72, as shown in Fig. 9.

**Fig. 10:**
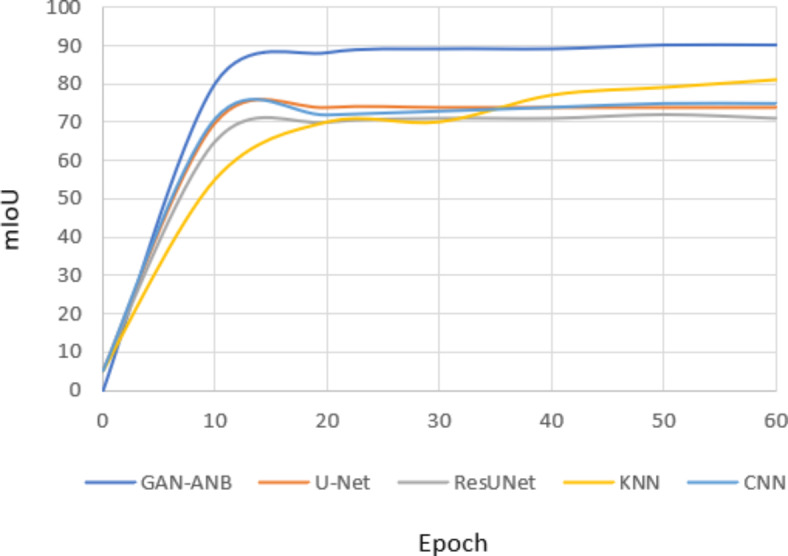
Comparison of mIoU.

GAN-ANB leads with an mIoU of 0.90, indicating its effectiveness in identifying the actual positives. KNN, CNN, and U-Net followed with mIoU values of 0.81, 0.75, and 0.74, respectively. ResUNet had the lowest recall of 0.71, as shown in Fig. 10.

**Fig. 11:**
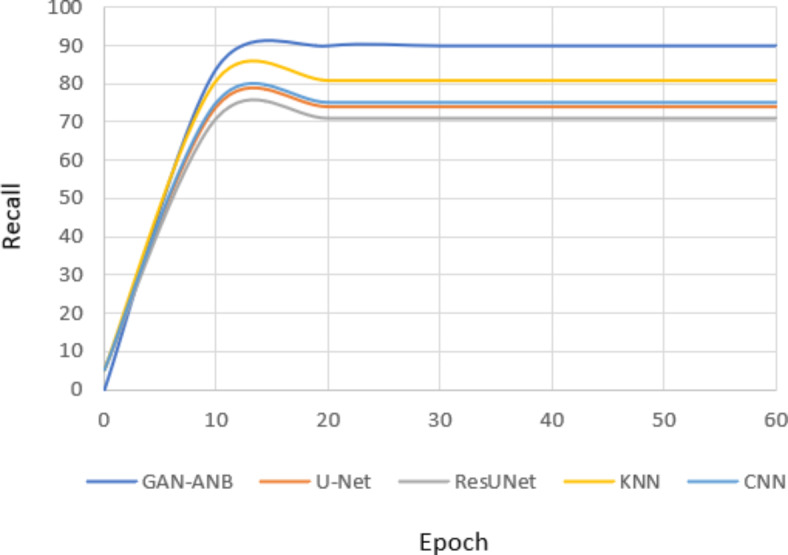
Comparison of Recall.

GAN-ANB showed the best recall with a value of 0.96. This means that they had a high proportion of true positives among the predicted positives. CNN, U-Net, and KNN had similar recall values of 0.78, 0.77, and 0.76, respectively. ResUNet again scored the lowest, with a recall of 0.74, as shown in Fig. 11.

**Fig. 12:**
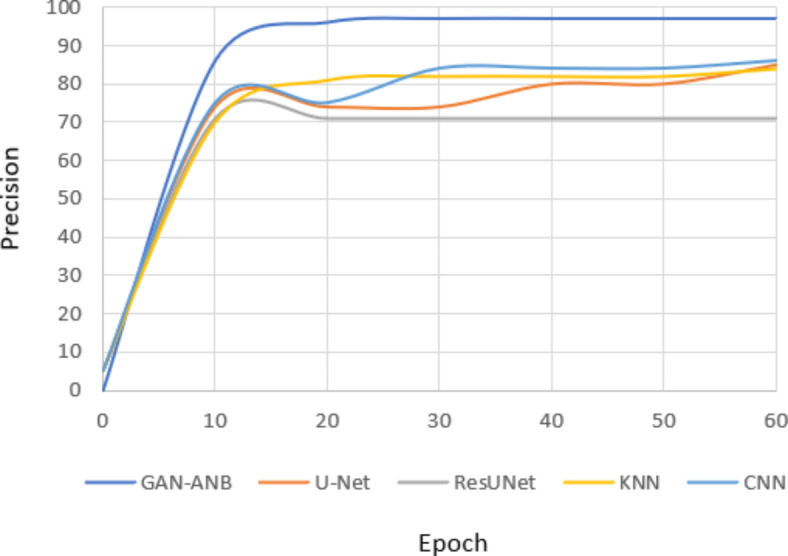
Comparison of Precision.

 GAN-ANB achieved the highest precision of 0.98, indicating a high proportion of correctly predicted pixels. CNN (0.86), U-Net (0.85), and KNN (0.84) followed closely, showing their robustness. ResUNet has the lowest precision at 0.83, indicating that it is slightly less reliable in terms of pixel-wise accuracy, as shown in Fig. 12.

**Fig. 13:**
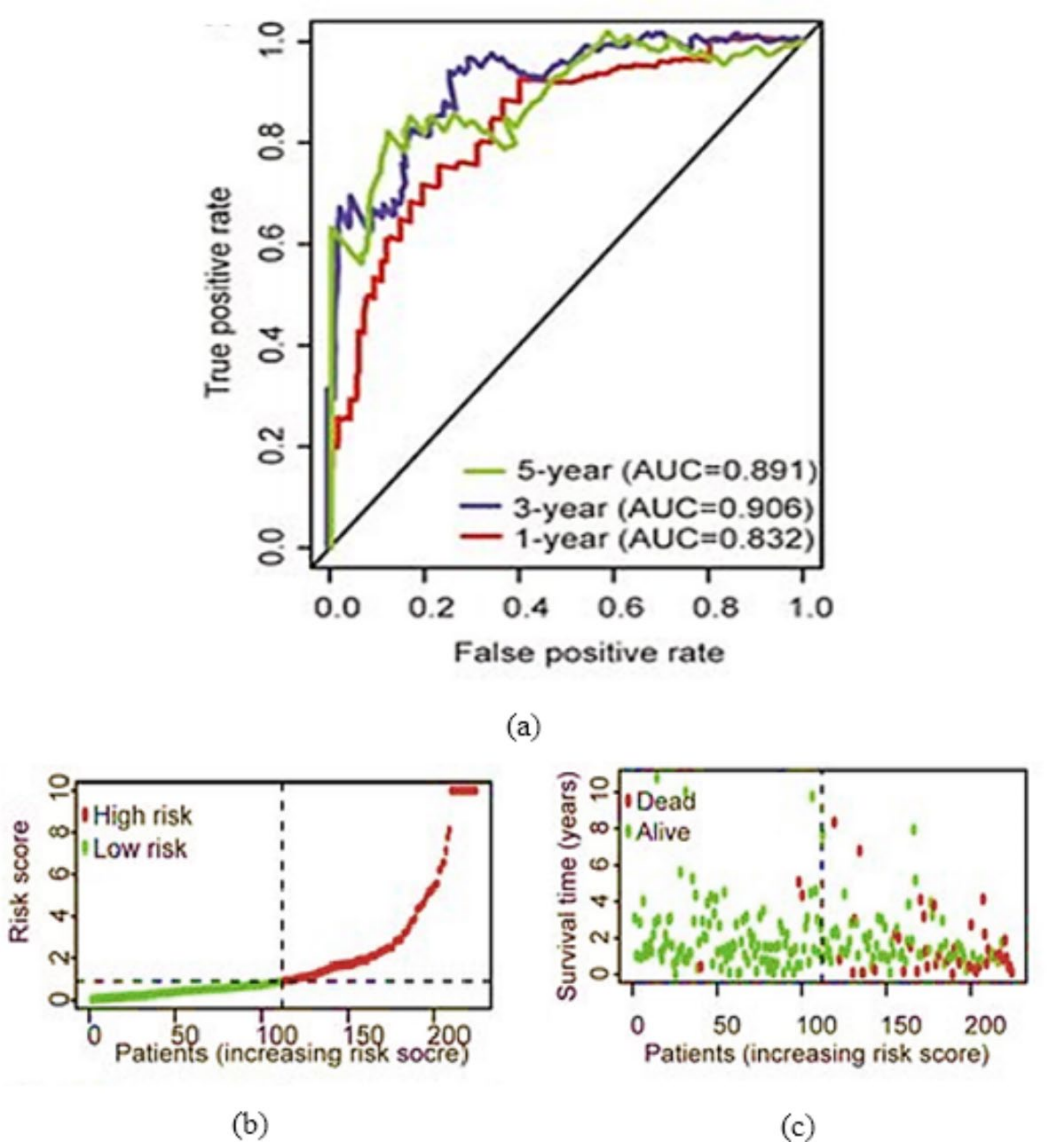
ROC of the proposed technique for classification of CVD.

The relationship between the True Positive Rate (TPR), also referred to as sensitivity, and the False Positive Rate (FPR), often referred to as 100% specificity for various classes, was depicted by the Receiver Operating Characteristic (ROC) curve. The entire area under the ROC curve is used to assess the extent to which the categorization procedure worked. The ROC curves and related AUC are shown in Fig. 13 to help visualize the classifier’s performance. Overall, the naÃ ¯ ve Bayes classification approach performed well. The 1-year, 3-year, and 5-year Overall Survival (OS) forecasts had AUC (Area Under the Curve (AUC) values of 0.89, 0.91, and 0.83, respectively, as shown in Fig. 13(a). In terms of predicting survival at three years (AUC = 0.91) and five years (AUC = 0.89), the model performed quite well. As shown in Fig. 13(b), the patient fatality rate increases in tandem with the risk score. With a p-value < 0.0001, the high-risk group had considerably worse OS than the low-risk group, as shown in Fig. 13(c).

## Conclusions

A potential method for detecting Coronary Computed Tomography Angiography (CCTA) imaging data-derived high-risk patients for Coronary Artery Disease (CAD) is to integrate Generative Adversarial Networks (GANs) with a generative adversarial network-augmented naïve Bayes (GAN-ANB) classifier. Through the creation of artificial CCTA images, GANs enrich the dataset and may improve the classification accuracy by capturing subtle patterns and characteristics that are essential for CAD risk assessment. In addition to improving the model’s generalization and robustness, the interaction between GANs and GAN-ANB classification provides the possibility of using cutting-edge image processing methods for CVD diagnostics. Through more precise and timely detection of high-risk CAD patients based on thorough imaging data analysis, this research path holds great promise for the development of individualized therapy. Future research should focus on validating the model across diverse populations and incorporating it into clinical decision support systems for real-time risk assessment and management.

## Data Availability

The datasets generated or analyzed during the current study are available from the corresponding author upon reasonable request.
